# Risk of serious infections during rituximab, abatacept and anakinra treatments for rheumatoid arthritis: meta-analyses of randomised placebo-controlled trials

**DOI:** 10.1136/ard.2007.083188

**Published:** 2008-01-18

**Authors:** C Salliot, M Dougados, L Gossec

**Affiliations:** René-Descartes University, Medicine Faculty; AP-HP, Cochin Hospital, Rheumatology B Department, Paris, France

## Abstract

**Background:**

Tumour necrosis factor α blockers in rheumatoid arthritis are known to increase the risk of serious infections defined as life-threatening, requiring hospitalisation or intravenous antibiotics. Recently, new biological agents have become available. Their safety is an important issue.

**Purpose:**

To assess if biological agents, ie rituximab, abatacept and anakinra increase the risk of serious infections in patients with rheumatoid arthritis in published randomised controlled trials.

**Data source:**

A systematic review of the literature using PUBMED, EMBASE, Cochrane library and abstracts databases (American College of Rheumatology and European League Against Rheumatism annual meetings) was performed up to October 2007. This search was completed with data from the Food and Drug Administration, the European Agency for the Evaluation of Medicinal Products and manufacturers.

**Data extraction:**

Three fixed-effect meta-analyses were performed to compare serious infection rates between each biological agent and placebo. Pooled odds ratios (ORs) were calculated, using the Mantel–Haenszel method with a continuity correction.

**Data synthesis:**

Twelve randomised controlled trials with data concerning serious infections were analysed (three for rituximab, five for abatacept and four for anakinra). They included 745 patients, 1960 patients, 2062 patients and 2112 patients treated by rituximab, abatacept, anakinra and placebo respectively. The overall pooled ORs did not reveal a statistically significant increased risk of serious infection for abatacept and rituximab; this risk was increased for high doses of anakinra (⩾100 mg daily) versus low dose and placebo (ORs = 9.63 (95% CI, 1.31 to 70.91) and 3.40 (95% CI, 1.11 to 10.46) respectively).

**Conclusions:**

These meta-analyses did not reveal a significant increase in the risk of serious infections during rituximab or abatacept treatments in patients with rheumatoid arthritis; however, high doses of anakinra may increase this risk, especially when patients have comorbidity factors. Large studies must be performed to confirm this safety profile in daily practice.

Rheumatoid arthritis (RA) is a systemic autoimmune disorder characterised by chronic polyarticular synovial inflammation that may lead to irreversible joint damage with disability and deformity. This joint inflammation is a result of the excessive production by activated T cells of pro-inflammatory cytokines, such as tumour necrosis factor (TNF) α, interleukin (IL)-1, IL-6, and the stimulation of immunoglobulin production by B cells.

The conventional treatment of RA combines corticosteroids and disease-modifying anti-rheumatic drugs (DMARDs), in particular, methotrexate. However, RA may remain active despite such treatments. Since 1997, new treatments based on biological agents have demonstrated their efficacy in RA. Biotherapies have different therapeutic targets and some are aimed against pro-inflammatory cytokines: three TNF-α blockers are available, infliximab, etanercept and adalimumab^1^^–^7 and one IL-1 receptor antagonist, anakinra.^8^ Down-regulation of T cell activation is achieved by the recombinant human fusion protein CTLA-4-immunoglobulin G (abatacept)^9^ and B cells are the selective target of the chimeric anti-CD20 monoclonal antibody (rituximab).^10^

Before the biotherapy era, it was reported that the incidence rate of infections in the RA population was nearly twice as high as in matched non-RA controls.^11^ This is thought to be related to the disease itself, which alters immunological functions, decreases mobility and causes skin defects, and also to immunosuppressive drugs, in particular concomitant use of steroids.^11^ ^12^ In post-marketing surveillance and observational studies of TNF-α blockers, serious infections (defined as life-threatening or requiring intravenous antibiotics or hospitalisation) appear to be the most frequent adverse event with a prevalence of 6–18% and an incidence rate of approximately 6 per 100 patient-years.^13^^–^15 Furthermore, case–control studies, conducted in routine daily practice, showed that the risk of serious infections was two- to three-fold higher in patients receiving TNF-α blockers compared with those not treated with such treatment.^13^^–^16 Thus it is clear that TNF-α blockers can increase immunosuppression in patients with RA and induce the emergence of serious infections. Meta-analysis is an interesting method to detect such a risk of a relatively rare event: a recent meta-analysis of randomised placebo-controlled trials of monoclonal anti-TNF-α antibodies (infliximab, adalimumab) found a pooled odds ratio (OR) for serious infections of 2.0 (95% confidence interval (CI), 1.3 to 3.1) in TNF-α blocker treated patients.^17^ However, individually, the trials had failed to demonstrate this increased risk of serious infections.

For other biological agents that may interfere with the immune response (rituximab, anakinra, abatacept), data on serious infections are lacking. The purpose of this study was to assess if these biotherapies increased the risk of serious infections in patients with RA, by performing a meta-analysis of data published to date.

## METHODS

For each biological agent, a meta-analysis was conducted according to the Cochrane Collaboration guidelines.^18^

### Study selection

A systematic literature search of the literature published up to December 2007 was performed in PUBMED, EMBASE and Cochrane library databases; without limitation of years of publication or journal, using the followings key-words: “rheumatoid arthritis”, “abatacept”, “rituximab”, “anakinra”, “clinical controlled trials”, “clinical trials”, “randomised controlled trials”, “clinical trials phase II, III, IV”. We also included congress abstracts of American College of Rheumatology (ACR) and European League Against Rheumatism (EULAR) meetings from 2004 to 2006, because we assumed that any abstract published prior to 2004 had been published in a formal full-length work. Moreover, to complete our search with unpublished data, the Food and Drug Administration (FDA), the European Agency for the Evaluation of Medicinal Products (EMEA) and the manufacturers (Roche, Amgen and Bristol-Myers Squibb) were contacted.

The trials were initially selected on the basis of their titles and abstract. The inclusion criteria were randomised placebo-controlled trials in adult patients with RA according to ACR criteria.^19^ The publications had to be written in English, French or Spanish. The patients had to be randomised to receive placebo or one of the three biological agents (rituximab, anakinra and abatacept), as monotherapy or with concomitant biological or non-biological DMARDs. Reviews and articles reporting trials that were not placebo-controlled were excluded.

### Data collection

One investigator (CS) selected the articles and collected the data, using a predetermined form. The following methodological features were collected: simple or double blinding, intention-to-treat-analysis or not, number of participants who completed the follow-up. For each trial, demographic characteristics (percentage female, mean age), RA features and its duration, treatment allocation (with doses and duration), concomitant treatments (DMARDs, corticosteroids, non-steroid anti-inflammatory drugs) and duration of follow-up were collected. In the literature, a serious infection is usually defined as life-threatening, requiring intravenous antibiotics or hospitalisations.^1^ ^5^ ^6^ This definition was also used here; if another definition of serious infections was given in the trials, it was also recorded. The number of patients with at least one serious infection in placebo and biological agent groups were collected. When available, the characteristics of infections (localisation, organism and outcome) were noted.

### Statistical analysis

For each biological agent, a fixed-effects meta-analysis of dichotomous outcomes was performed. This model has a superior performance to random-effect models when pooling trials with few or no events, such as serious infections.^20^ Because serious infections were rare events and some trials were small, the Mantel–Haenszel method was chosen with a continuity correction when there was no serious infection observed in one study arm of a trial. The Mantel–Haenszel method was used to estimate the pooled OR, with 95% CI, of all trials, assuming a fixed-effect model. The CIs for ORs were evaluated using the Robins, Breslow and Greenland variance formula and a χ^2^ test was given with its associated probability that pooled ORs were equal to 1. These statistical methods applied to each biological agent and for all trials whatever the dose, and for two a priori empirically pre-defined dose groups: low-dose group (500 mg for rituximab, ⩽2 mg/kg for abatacept and <100 mg for anakinra) and high-dose group (1000 mg for rituximab, 10 mg/kg for abatacept and ⩾100 mg for anakinra). We also performed a sensitivity analysis concerning potential confounding factors of infections. According to Doran *et al*^21^ predictive factors of serious infections in patients with RA were increasing age, presence of rheumatoid factor (RF), extra-articular manifestations, nodules, increased erythrocyte sedimentation rate, comorbidities (such as diabetes mellitus, chronic lung disease, alcoholism, organic brain disease, leucopenia) and concomitant steroid treatment. When available, these factors were included in the sensitivity analysis. According to this study, duration of RA, previous and concomitant DMARDs, sex, obesity and smoking status did not appear as predictors of serious infections in patients with RA. We used StatsDirect version 2.5.7 (StatsDirect Ltd, Cheshire, UK) and RevMan version 4.2 (Review Manager, Copenhagen, The Nordic Cochrane Centre, 2003) statistical software.

## RESULTS

### Literature search results and trials characteristics

**Figure 1:**
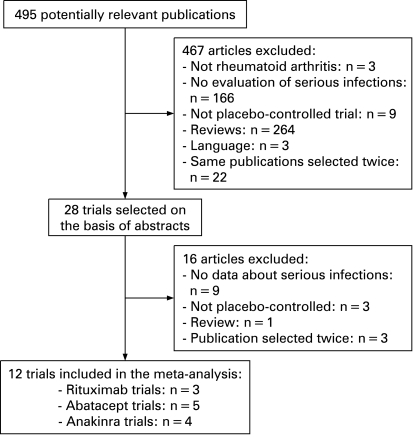
Systematic literature search selection process.

Figure 1 shows the selection process of published trials for this systematic review. Initially, 490 potentially relevant articles were screened. Among them 477 were excluded. Finally, 13 randomised double-blind placebo-controlled trials were included.^8^^–^10 22^–^31 The characteristics of these trials are summarised in table 1. Twelve published trials were selected for the meta-analysis. One article was excluded from the meta-analysis because it reported a trial already selected, with a different follow-up:^24^ ^25^ for the meta-analysis, we selected the longest follow-up (48 vs 24 weeks). No additional randomised controlled trial was available from FDA, EMEA, manufacturers or congress abstracts.

**Table 1:** Characteristics of the 12 randomised controlled trials of biotherapies in RA included in these meta-analyses for serious infections

Sources (reference)	No. of randomised patients (no. of patients treated*)	No. who completed the follow-up	RA characteristics	Study protocol with doses (no. of patients treated*in each group)	Duration of follow-up (weeks)
Rituximab					
Edwards *et al*, 2004 10	161 (161)	130	Active RA despite MTX	⩾10 mg per week of MTX+placebo (40) 1000 mg on days 1 and 15 of rituximab (40) 1000 mg on days 1 and 15 of rituximab+750 mg on days 3 and 17 of cyclophosphamide (41) 1000 mg on days 1 and 15 of rituximab+ ⩾10 mg per week of MTX (40)	48
Emery *et al*, 2006 22	465 (465)	375	Active RA resistant to DMARDs, including biological agents	10–25 mg per week of MTX+placebo (149) 1000 mg on days 1 and 15 of rituximab+ 10–25 mg per week of MTX (192) 500 mg on days 1 and 15 of rituximab+ 10–25 mg per week of MTX (124)	24
Cohen *et al*, 2006 23	520 (517)	366	Active RA despite TNF-α blockers	10–25 mg per week of MTX+placebo (209) 10–25 mg per week of MTX+1000 mg on days 1 and 15 of rituximab (308)	24
Abatacept					
Moreland *et al*, 2002 9	122 (122)	90	Refractory RA despite DMARDs or etanercept	Placebo (32) 0.5 mg/kg of abatacept on days 1, 15, 29 and 57 (26) 2 mg/kg of abatacept on days 1, 15, 29 and 57 (32) 10 mg/kg of abatacept on days 1, 15, 29 and 57 (32)	12
Kremer *et al*, 2003 and 2005 24,25	339 (339)	235	Active RA despite MTX	10–30 mg per week of MTX+placebo (119) 10–30 mg per week of MTX+abatacept 2 mg/kg on days 1, 15 and 30 and every 30 days thereafter (105) 10–30 mg per week of MTX+abatacept 10 mg/kg on days 1, 15 and 30 and every 30 days (115)	48
Genovese *et al*, 2005 26	393 (391)	322	Active RA despite at least 3 months of TNF-α blocker	DMARDs+placebo (133) DMARDs+10 mg/kg of abatacept on days 1, 15, 29 and every 28 days (258)	24
Kremer *et al*, 2006 27	656 (652)	547	Active RA despite MTX	⩾15 mg per week of MTX+placebo (219) ⩾15 mg per week of MTX+10 mg/kg of abatacept on days 1, 15 and 29, and every 28 days (433)	48
Weinblatt *et al*, 2006 28	1456 (1441)	1231	Active RA despite biological or non-biological DMARDs	At least 1 non-biological DMARD+placebo (418) 1 biological DMARD+placebo (64) At least 1 non-biological DMARD+10 mg/kg of abatacept on days 1, 15 and 29 and every 4 weeks thereafter for a total of 14 infusions (856) 1 biological DMARD+10 mg/kg of abatacept on days 1, 15 and 29 and every 4 weeks thereafter for a total of 14 infusions (103)	48
Anakinra					
Bresnihan *et al*, 1998 8	472 (472)	468	Active and severe RA	Placebo (121) 30 mg daily of anakinra (119) 75 mg daily of anakinra (116) 150 mg daily of anakinra (116)	24
Cohen *et al*, 2002 29	419 (419)	331	Moderate to severe active RA despite MTX	MTX (15–25 mg/wk)+placebo (74) MTX (15–25 mg/wk)+daily 0.04 or 0.01 or 0.4 or 1 or 2 mg/kg of anakinra (345)	24
Cohen *et al*, 2004 30	506 (501)	492	Active RA despite MTX	MTX (10–25 mg/wk)+placebo (251) MTX (10–25 mg/wk)+100 mg per day of anakinra (250)	24
Schiff et al, 2004† 31	1414 (1399)	1105	Active RA with and without comorbidity factor	DMARDS+placebo (283) DMARD+100 mg per day of anakinra (1116)	24

RA, rheumatoid arthritis; MTX, methotrexate; DMARDs, disease-modifying antirheumatic drugs; wk, week.

*Number of patients who received at least one dose of study medication in this arm of randomisation and were analysed.

†Including 951 patients with comorbidity factors (775 in the anakinra group and 196 in the placebo group).

All of these 12 articles were randomised double-blind placebo-controlled trials with a follow-up of 12–48 weeks, for patients with RA according to the ACR criteria and with active disease despite DMARDs. However, three studies concerned patients with RA refractory to TNF-α blocker treatment.^9^ ^22^ ^26^ In all trials, an intention-to-treat analysis was performed and 94% of the patients included completed the follow-up. Intention-to-treat analysis included all randomised patients who received at least one dose of study medication (modified intention-to-treat analysis). Thus a total of 4767 patients received at least one dose of one of the three biological agents and 2112 placebo. Eighty-one per cent of the participants were women with a mean age at inclusion of between 46 and 57 years. The mean duration of RA was 9.2 years (range 3.4–12.1).

### Serious infections

According to these trials, serious infection was defined as life-threatening, fatal, requiring a hospitalisation, intravenous antibiotics, or resulting in persistent or significant disability. Tables 2 and 3 summarise the serious infections reported in the 12 trials, according to treatment groups with incidence and ORs.

**Table 2:** Summary of serious infections* in the 12 randomised placebo-controlled trials included in these meta-analyses

Treatment	No. of trials(references)	No. of patients treated† (biological/placebo groups)	Duration of follow-up (weeks)	No. of patients with at least 1 serious infection in biotherapy groups and by dose group(no. of participants)	No. of patients with at least 1 serious infection in placebo groups(no. of participants)
Rituximab	3 10,22,23	1143 (745/398)	24–48	0 Rituximab 500 mg (124)	6 (398)
				17 Rituximab 1000 mg (621)	
Abatacept	5 9,24^–^28	2945 (1960/985)	24–48	0 Abatacept 0.5 mg/kg (26)	18 (985)
				2 Abatacept 2 mg/kg (137)	
				47 Abatacept 10 mg/kg (1797)	
Anakinra	4 8,29^–^31	2771 (2062/729)	24	0 Anakinra 0.04 mg/kg (63)	4 (729)
				0 Anakinra 0.1 mg/kg (74)	
				0 Anakinra 0.4 mg/kg (77)	
				0 Anakinra 1 mg/kg (59)	
				0 Anakinra 2 mg/kg (72)	
				0 Anakinra 30 mg per day (119)	
				1 Anakinra 75 mg per day (116)	
				25 Anakinra 100 mg per day (1367)	
				4 Anakinra 150 mg per day (116)	

*Serious infection was defined as life-threatening, requiring intravenous antibiotics or hospitalisation.

†Number of patients who received at least one dose of study medication in this arm of randomisation and were analysed.

**Table 3:** Risk of serious infections in patients with RA during rituximab, abatacept and anakinra treatments in randomised placebo-controlled trials

Source	Treatment	No. of patients with at least 1 serious infection/total in treatment groups(incidence %)	No. of patients with at least 1 serious infection/total in placebo groups(incidence %)	Pooled ORs* (95% CI)
Edwards *et al*, 2004	Rituximab	6/121 (4.9)	1/40 (2.5)	1.45 (0.56 to 3.73)
Emery *et al*, 2006		4/316 (1.2)	2/149 (1.3)	
Cohen *et al*, 2006		7/308 (2.3)	3/209 (1.4)	
Total		17/745 (2.3)	6/398 (1.5)	
Moreland *et al*, 2002	Abatacept	1/90 (1.11)	0/32 (0)	1.35 (0.78 to 2.32)
Kremer *et al*, 2005		1/220 (0.45)	2/119 (1.7)	
Genovese *et al*, 2005		6/258 (2.3)	3/133 (2.2)	
Kremer *et al*, 2006		13/433 (3.0)	5/219 (2.3)	
Weinblatt *et al*, 2006		28/959 (2.9)	8/482 (1.6)	
Total		49/1960 (2.5)	18/985 (1.8)	
Bresnihan *et al*, 1998	Anakinra	5/351 (1.42)	1/121 (0.82)	2.75 (0.90 to 8.35)
Cohen *et al*, 2002		0/345 (0)	0/74 (0)	
Cohen *et al*, 2004		2/250 (0.80)	2/251 (0.8)	
Schiff *et al*, 2004		23/1116 (2.0)	1/283 (0.3)	
Total		30/2062 (1.4)	4/729 (0.5)	

*Using the Mantel–Haenszel method to calculate a pooled ORs with fixed effect.

χ^2^ (test OR differs from 1): for Rituximab: χ^2^ = 0.29, p = 0.6, for Abatacept: χ^2^ = 0.94, p = 0.3, for Anakinra: χ^2^ = 2.5, p = 0.1.

### Serious infections during rituximab treatment

**Figure 2:**
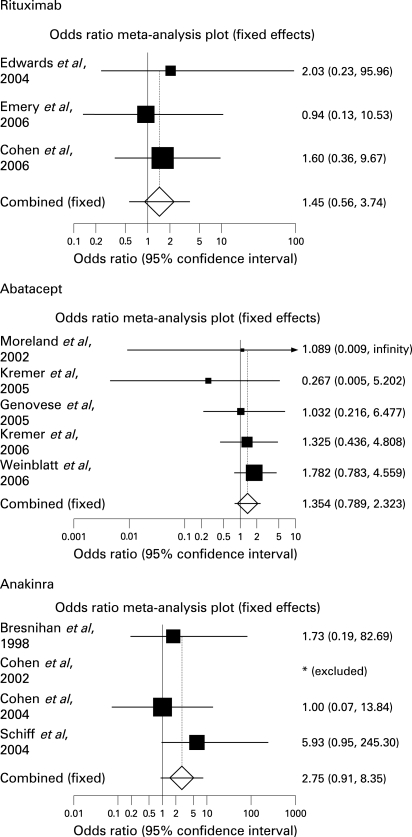
Effect of biological agents (rituxumab, abatacept and anakinra) versus placebo on serious infections (Forest plot).

Three trials fulfilled the criteria for analysis^10^ ^22^ ^23^ (table 1). Overall, 745 randomised patients with RA received at least one dose of rituximab (500 mg or 1000 mg) and 398 received at least one dose of placebo. The mean age of patients receiving rituximab was 52.0 years. Among them, 490 had steroids as concomitant treatment (65.7%) and 331 (among 624 patients tested) were RF positive (82.5%). For patients allocated to placebo, the mean age was 52.3 years, 253 had steroid treatment (63.5%) and 207 (among 358 patients tested) were RF positive (83.6%). Six and 17 serious infections were observed in the placebo and rituximab groups respectively. These 17 serious infections occurred in patients receiving 2×1000 mg infusions of rituximab. No serious infections were observed in those treated with the dose of 500 mg (table 2). The incidence of serious infections was 2.3% and 1.5% in the rituximab and placebo groups respectively (table 3). Using the Mantel–Haenszel method, the overall pooled OR for serious infections whatever the dose and according to the dose groups were not significantly increased (tables 3 and 4, fig 2). In patients receiving rituximab, serious infections were mainly respiratory tract bacterial infection. Among the 17 patients who had one serious infection: five had bronchopneumonia (one of them presented with two episodes of *Pseudomonas aeruginosa* pneumonia), two septic arthritis (of whom one *Staphylococcus aureus* septicaemia), three pyelonephritis and two gastroenteritis and one each epiglottitis, cellulitis of a toe and acute hepatitis B. One fatal bronchopneumonia occurred in a patient receiving rituximab.^10^ No opportunistic infection or tuberculosis occurred in patients receiving rituximab.

**Table 4:** Risk of serious infections stratified by high- and low-dose dose groups

Treatment	ORs (95% CIs)		
	High-dose* versus placebo groups	Low-dose† versus placebo groups	High-dose* versus low-dose† groups
Rituximab	1.68 (0.64 to 4.35)	0.24 (0.01 to 4.33)	7.20 (0.43 to 120.66)
Abatacept	1.35 (0.78 to 2.33)	0.84 (0.13 to 5.30)	2.16 (0.52 to 8.98)
	1.24 (0.70 to 2.29)‡		2.0 (0.48 to 8.33)‡
Anakinra	3.40 (1.11 to 10.46)	0.51 (0.03 to 8.27)	9.63 (1.31 to 70.91)
	1.67 (0.51 to 5.41)§		6.41 (0.81 to 50.30)§

*High-dose groups were defined as 1000 mg for rituximab, 10 mg/kg for abatacept and ⩾100 mg for anakinra.

†Low-dose groups were defined as 500 mg for rituximab, ⩽2 mg/kg for abatacept and <100 mg for anakinra.

‡Calculated ORs when patients receiving biological DMARD as concomitant treatment were excluded.

§Calculated ORs when patients with comorbidity factors were excluded.

### Serious infections during abatacept treatment

Five published placebo-controlled trials fulfilled the selection criteria for analysis^9^ ^25^^–^28 (table 1). A total of 2945 randomised patients received at least one dose of abatacept (0.5, 2 or 10 mg/kg) (n = 1960) or placebo (n = 985) for a duration of treatment comprised between 24 and 48 weeks. One hundred and three participants treated with abatacept and 64 patients receiving placebo had TNF-α blocker or anakinra as concomitant treatment.^28^ In the abatacept groups, the mean age was 49.6 years, 1328 patients received steroids as concomitant treatment (67.7%) and 82.5% (among the 872 patients tested) were RF positive. Concerning placebo groups, the mean age was 48.6 years, 655 patients took concomitant steroid treatment (66.5%) and 79.8% (among the 471 patients tested) were RF positive.

In the abatacept group, 49 serious infections were observed versus 18 in the placebo group (tables 2 and 3). Seven serious infections (six in abatacept and one in placebo groups) occurred in patients who received TNF-α blocker or anakinra as concomitant treatment.^28^ Thus, the incidence was 2.5% and 1.7% respectively with an overall pooled OR whatever the dose and according to the dose groups not significantly increased (tables 3 and 4, fig 2). The 49 serious infections occurring with abatacept were mainly bronchopulmonary,^23^ streptococcal and pyogenic septicaemia,^2^ staphylococcal arthritis,^2^ abscesses,^2^ gastrointestinal (six of whom three diverticulitis), dermatological infections (six of whom one was a cellulitis) and pyelonephritis.^7^ One case of unconfirmed tuberculosis and one case of pulmonary aspergillosis were reported. The last patient (who had a history of tuberculosis and pulmonary fibrosis) died of aspergillosis and of a *Pseudomonas aeruginosa* septicaemia.

### Serious infections during anakinra treatment

Four trials were included in the meta-analysis.^8^ ^29^^–^31 Their characteristics are summarised in table 1. The meta-analysis was performed on 2771 patients, randomised to receive at least one dose of anakinra (n = 2062) (0.04, 0.01, 0.4, 1, 2 mg/kg, 30, 75, 100 or 150 mg) or placebo (n = 729), during 24 weeks. Among these participants, 755 and 196 patients (who received anakinra and placebo respectively) had comorbidity factors such as pulmonary chronic disease, diabetes, renal impairment, previous malignancy or infection, cardiovascular or central nervous system diseases.^31^ The proportion of patients with comorbidity factors was similar in the anakinra and placebo groups (67.6% and 69.2% respectively). In the anakinra groups, the mean age was 54.2 years and 53.0%% received steroids as concomitant treatment. Concerning placebo groups, the mean age was 55.3 years and 55.0% received steroids. The prevalence of RF was 74.6% in the anakinra groups and 75.1% in placebo groups (for 946 and 446 patients tested respectively). Thirty serious infections (1.4%) were observed in the anakinra group versus 4 (0.5%) in placebo group (tables 2 and 3). Nineteen serious infections (2.5%) occurred in patients with comorbidity factors and treated with anakinra.^31^ The overall pooled OR of serious infections did not show a significantly increased risk of serious infection. However, the risk was increased for a high dose of anakinra versus low dose and high dose versus placebo (ORs = 9.63 (95% CI: 1.31 to 70.91) and 3.40 (95% CI: 1.11 to 10.46) respectively) (tables 3 and 4, fig 2). When patients with comorbidity factors were excluded, these results were not statistically significant whatever the dose groups (table 4). Among the 30 serious infections occurring in anakinra-treated groups, 11 were pneumonia. The others were osteomyelitis,^2^ cellulitis,^3^ bursitis, herpes zoster, infected bunion and gangrene (one of each). No related death or opportunistic infections were described.

### Sensitivity analyses

Analyses of subgroups according to age (< or > to median, 52.7 years), concomitant intake of steroids (median 65% of patients) and RF positivity (median positivity 78% of patients), confirmed the previous results (data not shown). Thus these potential predictive factors of serious infections did not appear as confounding factors.

## DISCUSSION

These meta-analyses of published data did not evidence an increased risk of serious infections for patients with RA treated by rituximab or abatacept. However, results indicated a significantly increased risk of serious infections for the high dose of anakinra.

The meta-analyses of randomised placebo-controlled trials that evaluated separately these three biotherapies included 12 trials: three for rituximab (745 patients treated), five for abatacept (1960 patients) and four for anakinra (2062 patients). Separately, these trials did not show an increased risk of serious infections except for one concerning patients with comorbidities receiving anakinra.^31^ The calculated pooled ORs, whatever the doses, did not show a significantly increased risk of serious infections in patients treated with these three biological agents. Indeed, overall ORs were 1.45 (95% CI, 0.56 to 3.73), 1.35 (95% CI, 0.78 to 2.32) and 2.75 (95% CI, 0.90 to 8.35) for rituximab, abatacept and anakinra respectively (table 3). Nevertheless, when high-dose group of anakinra was compared with placebo and low-dose groups, the risk of serious infections was increased during anakinra treatment with ORs of 3.40 (95% CI, 1.11 to 10.46) and 9.63 (95% CI, 1.31 to 70.91) respectively. For high doses of rituximab and abatacept, we observed a tendency towards an increased risk during biological agent treatments versus low-dose groups: ORs were 7.20 (95% CI, 0.43 to 120.66) and 2.16 (95% CI, 0.52 to 8.98) respectively. Thus, although CIs include 1, there is a lingering concern for infectious risks with these drugs. The serious infections reported were in the majority bacterial and bronchopulmonary. Opportunistic infections seemed to be unusual. Indeed only two were described during abatacept treatment: one case of tuberculosis (unconfirmed) and one of pulmonary aspergillosis.

Randomised controlled trials are often not adapted to demonstrate an increased risk for rare side-effects because the numbers of patients analysed are too small and the exposure time too brief. Post-approval observational studies usually lack a control group, which means establishing causality between a treatment and an event is impossible. Therefore, to pool results of randomised controlled trials by meta-analysis, is an interesting and powerful alternative.^17^ ^19^

Incidences of serious infections observed in the trials analysed are close to other published incidences in RA. Among the 2112 patients receiving placebo, 28 had a serious infection. The incidence was 1.3% and close to the one observed in placebo groups of other controlled trials in patients with RA. Indeed, in the nine randomised controlled trials that evaluated anti-TNF antibodies, 26 serious infections were reported among 1512 patients receiving placebo (incidence 1.7%).^17^ High-dose anakinra (⩾100 mg) seemed to increase the risk of serious infections. This result concerned 1399 treated patients of whom 755 participants had at least one comorbid condition (such as cardiovascular, pulmonary, diabetes, a history of infection, renal impairment, etc.).^31^^–^33 Indeed, when we excluded from the meta-analysis the patients with comorbidity, the OR no longer showed a significantly increased risk in the high-dose anakinra group. Thus comorbidity factors appear to play a part in these results. The long-term safety of anakinra treatment was evaluated in an open-label study following the randomised phase. A total of 1346 patients received anakinra for up to 3 years.^34^ Serious infections were defined as infections necessitating hospitalisation or the use of intravenous antibiotics. The incidence rates (per 100 patient-years) of serious infections concerning patients receiving anakinra were 5.2 during the first placebo-controlled 6 months (versus 1.6 for placebo group) and 5.4 over the 36 months. The most frequent infections were pneumonia and cellulitis. Thus long-term use of anakinra appears to be safe in patients with RA. Nevertheless, this study showed that patients who received steroids as concomitant treatment at baseline were much more likely to experience a serious infection (7.13 per 100 patient-years in patients with steroids versus 2.87 per 100 patient-years for patients without). In this meta-analysis, we could not evaluate the interaction between corticosteroid and anakinra because individual data were not available, the present study cannot fully clarify the issue of an interaction between steroids and anakinra or the role of individual comorbidity factors.

This systematic review was performed using all available literature sources and includes all data published to date. Furthermore, the meta-analysis conforms to the recommendations of the Cochrane collaboration. Therefore we consider these results to be valid. However, this study does have shortcomings. Only published data were analysed, thus we cannot exclude the possibility of publication bias. If the reasons that studies remain unpublished are associated with their outcome, then the results of a meta-analysis could be biased.^38^ Furthermore, biases in patient selection may influence the final results, ie the exclusion from the trials of patients with comorbidities and previous serious infections. However, in four trials evaluating rituximab or abatacept, some patients had received previously one TNF-α blocker. In Genovese’s trial, all patients (391) were previously treated with one TNF-α blocker.^25^ These data lacked in Moreland *et al*’s trial.^9^ Concerning rituximab, 769 patients (of the 1143) received previously a TNF blocker.^22^ ^23^ It is possible that these patients were not at high risk of serious infection (we suppose that patients previously treated with TNF blockers were screened twice and that those who had a serious infection during the TNF blocker treatment were not included in another trial evaluating a biological agent).

These meta-analyses do not demonstrate an increased risk of serious infections with the biological agents analysed. One of the important issues with these results is the potential lack of power. Thus, for each biological agent we calculated a posteriori the number of patients that it would be necessary to analyse in treated and placebo groups to demonstrate the increase in risk that was observed, though it was non-significant, with a power of 80% (α risk = 5%). In each group, for rituximab, 4569 patients would be necessary, and 6737 for abatacept. Thus, these meta-analyses are underpowered to detect the observed magnitude of difference in infection risk for these two biological agents. However, for anakinra, the number of patients necessary was 1820 in each group. This meta-analysis included a sufficient number of patients in the anakinra arm (2062) but only 729 patients in the placebo group, thus lack of power is less an issue for anakinra. Because of this lack of power, it is not possible to ascertain completely the absence of infectious risks due to the analysed biological agents.

Like TNF-α blockers, anakinra is an anticytokine biological agent. TNF-α blockers increase the risk of opportunistic infections in patients with RA, most frequently due to intracellular organisms. Tuberculosis, especially extrapulmonary and disseminated, was the most frequently reported granulomatous infection and occurred with the three TNF-α blockers.^34^^–^36 Invasive opportunistic infections occurring with the three TNF-α blockers have been reported, such as listeriosis, candidosis, histoplasmosis, nocardiosis, aspergillosis, pneumocystosis, etc.^39^^–^41 In the present meta-analysis, tuberculosis and opportunistic infections seem to be exceptional during rituximab, abatacept or anakinra treatments, thus demonstrating the difference in mechanisms of action between these biotherapies, especially against intracellular organisms.

According to these meta-analyses performed on the basis of randomised placebo-controlled trials, rituximab, abatacept and anakinra seem to be safe as regards the risk of serious infections. Nevertheless, an increased risk was observed for high doses of anakinra (⩾100 mg) in patients with comorbidity factors. Moreover, such clinical trials select patients who are not representative of all patients with RA in daily practice. Thus the use of these biological agents will require careful monitoring in daily practice especially in patients with comorbidity conditions and with concomitant treatments, such as steroids. Further, large-scale, post-marketing studies will have to confirm these data.
